# Novel α-N-heterocyclic thiosemicarbazone complexes: synthesis, characterization, and antimicrobial of properties investigation†

**DOI:** 10.1039/d4ra04002c

**Published:** 2024-09-16

**Authors:** Dilek Nartop, Elvan Hasanoğlu Özkan, Hatice Öğütcü, Nurdan Kurnaz Yetim, İnci Özdemir

**Affiliations:** a Department of Chemistry, Faculty of Arts and Sciences, Düzce University Düzce Turkiye; b Department of Chemistry, Faculty of Sciences, Gazi University Ankara Turkiye ehasanoglu@gazi.edu.tr; c Department of Field Crops, Faculty of Agriculture, Kırşehir Ahi Evran University Kırşehir Turkiye; d Department of Chemistry, Faculty of Arts and Sciences, Kırklareli University Kırklareli Turkiye; e Kocaeli University, Izmit Vocational School, Property Protection and Security Department 41285 Kocaeli Turkiye

## Abstract

In this paper, eight novel α-N-heterocyclic thiosemicarbazone complexes were synthesized in search of new biologically active compounds, and characterized *via* organic elemental analysis, nuclear magnetic resonance spectroscopy, infrared spectra, thermogravimetric analysis, ultraviolet-visible spectroscopy, molar conductance and magnetic susceptibility measurements. The *in vitro* antimicrobial activity of these complexes was examined against ten disease-causing pathogens: Gram-positive bacteria (*Micrococcus luteus* ATCC9341, *Staphylococcus epidermidis* ATCC12228, *Bacillus cereus* RSKK863) and Gram-negative bacteria (*Pseudomonas aeroginosa* ATCC27853, *Klebsiella pneumonia* ATCC27853, *Enterobacter aerogenes* ATCC51342, *Salmonella typhi* H NCTC9018394, *Shigella dysenteria* NCTC2966, *Proteus vulgaris* RSKK96026) and yeast (*Candida albicans* Y-1200-NIH). The results revealed that the α-N-heterocyclic thiosemicarbazone compounds showed potent activity. It was observed that all thiosemicarbazone complexes were more susceptible to Gram-negative strains based on the presence of an electron-withdrawing substituent (–Br/–Cl/–F). It was determined that thiosemicarbazone Cu^2+^complexes showed stronger antifungal effects.

## Abstract 2

In this paper, eight novel α-N-heterocyclic thiosemicarbazone complexes were synthesized in search of new biologically active compounds, and characterized *via*^1^H-NMR, IR, TGA, UV-Vis, molar conductance and magnetic susceptibility measurements.

## Graphical abstract

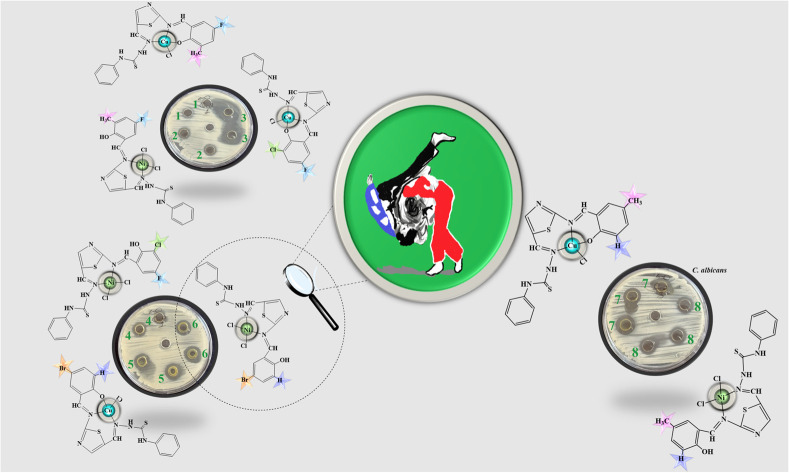

## 1 Introduction

Heterocyclic compounds are organic structures in which some carbon atoms are replaced by heteroatoms such as nitrogen, sulphur, oxygen, and phosphorus.^1^ Heterocyclics have different physical and chemical properties and reactivity depending on the heteroatoms' ring size and structure.^2^ Recently, there has been an increasing interest in heterocyclic compounds due to their various applications. The synthesis of innovative, stereo-selective, functional new heterocyclic compounds is of great interest for drug discovery and development.^3^ Heterocyclic compounds are used in the agrochemical industry in crop protection due to their pesticidal activities.^4^ Heterocycles are used in the pharmaceutical industry to treat some diseases (such as Alzheimer's, cancer, diabetes, circulatory diseases, and AIDS) due to their therapeutic properties.^5^ Heterocycles bind with enzymes due to their variety of intermolecular interaction properties. These properties of heterocycles are the reasons for preference in anti-cancer drug design.^6^ Particularly, thiosemicarbazone-based heterocyclics are an important class of heterocyclic compounds because of their coordination capacity. ^7^ They are important chelating ligands due to the containing potential donor atoms (deprotonated phenolic oxygen, thione/thiol sulphur, azomethine nitrogen, *etc.*).^8^ Especially, α-N-heterocyclic thiosemicarbazones, in which the thiosemicarbazone side chain is linked to an N-heterocyclic ring at the α position, are strong metal chelating agents.^9^ Heterocyclic compounds containing thiosemicarbazone have various biological, cytotoxic, and pharmacological activities. These properties of thiosemicarbazones are generally related to the presence of imine group (–N=CH–) and metal ion coordination. The coordination affects such properties as lipophilicity, drug resistance, *etc.*^10–12^ The complexes can exhibit bioactivities not shown by the free ligands.^13^ Heterocyclic thiosemicarbazones and their metal complexes exhibit a broad range of activities such as antibacterial, antioxidant, antitumor, antimicrobial, antiviral, antifungal, antimalarial, anticonvulsant, anti-HIV, antiamoebic, antiproliferative, anti-inflammatory, antidiabetic, and anticancer.^14–18^ Heterocyclic thiosemicarbazone complexes of nickel metal play an important role in the biology of microorganisms and plants, have variable binding modes, and show strong biological, antibacterial, and inhibitory activity.^10,19–22^ Heterocyclic thiosemicarbazone complexes of copper, which catalysed redox reactions and are essential for life and the most effective available divalent ion for binding to organic compounds, strong exhibit antibacterial, antitumor, anticancer, and antimicrobial activity.^23–26^ Thiosemicarbazone transition metal complexes have been reported as extensively effective and preferred drugs.^27,28^ The synthesis of new heterocyclic thiosemicarbazone metal complexes is important due to their biological and pharmacological properties. Therefore, novel α-N-heterocyclic thiosemicarbazone complexes were synthesized using thiosemicarbazide, carboxaldehyde, aldehyde derivatives, and metal salts.

It is predicted that the new heterocyclic thiosemicarbazone Ni^2+^ and Cu^2+^ complexes synthesized within the scope of this study will contribute to the importance of heterocyclic chemistry in the pharmaceutical industry and medicinal chemistry.

## 2 Experimental

### 2.1. Materials and measurements

All chemicals were procured from Sigma-Aldrich. Organic elemental analyses were obtained with a Thermo-Scientific Flash-2000 model elemental analyzer. IR spectra were recorded from KBr pellets using a Shimadzu IR Prestige-21 model spectrometer. ^1^H-NMR spectra were measured on a Bruker Biospin brand Avance III 400 MHz model device. UV-Vis spectra were determined on a UV-1800 ENG240V, Soft model spectrophotometer. TGA analysis were carried out using a Shimadzu DTG 60H-DSC 60 model thermal analyzer. The molar conductance of complexes was measured in dimethyl sulphoxide (DMSO) at 21 °C on Conductivity 430 Lab. Magnetic measurements were obtained with a Sherwood Scientific MKI model Evans magnetic susceptibility device.

### 2.2. Synthesis of heterocyclic compounds containing thiosemicarbazone

**Fig. 1:**
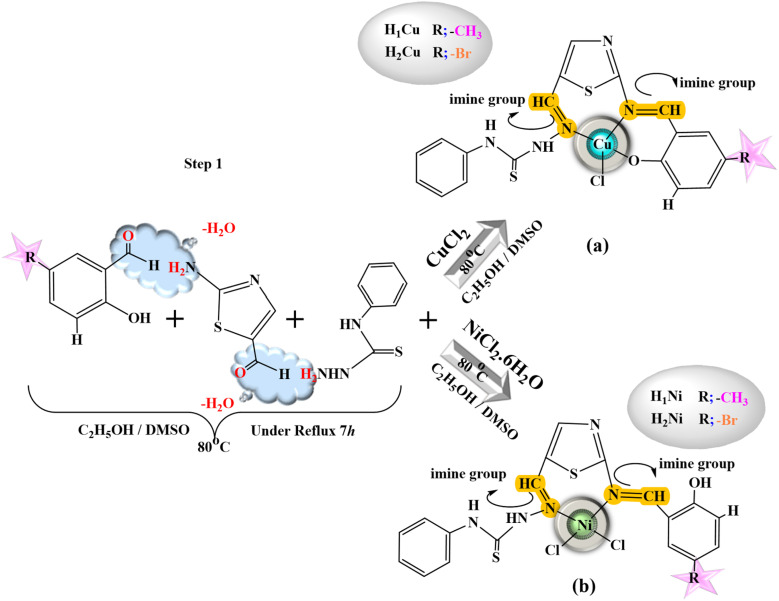
General procedure for the heterocyclic thiosemicarbazone (a) Cu complexes (H_1_Cu, H_2_Cu) and (b) Ni complexes (H_1_Ni, H_2_Ni).

**Fig. 2:**
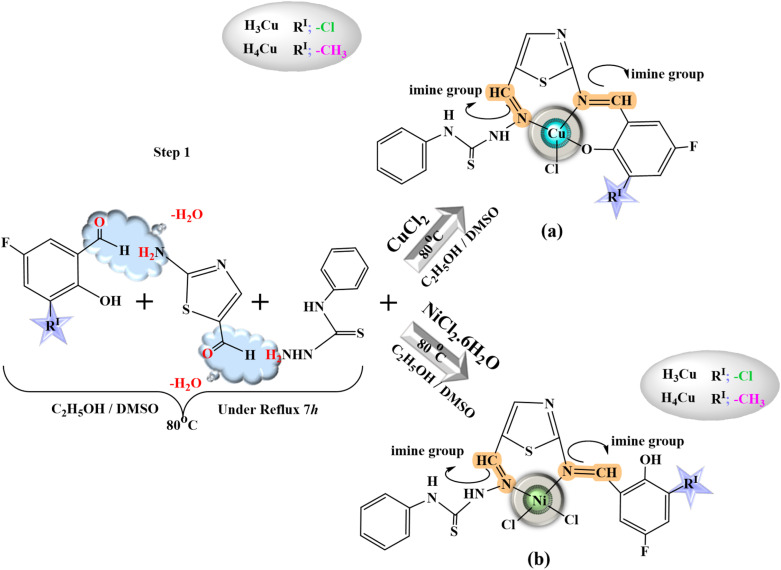
General procedure for the heterocyclic thiosemicarbazone (a) Cu complexes (H_3_Cu, H_4_Cu) and Ni complexes H_3_Ni, H_4_Ni).

All heterocyclic thiosemicarbazone Ni^2+^ and Cu^2+^ complexes were prepared with the template method (Fig. 1 and 2). Novel heterocyclic thiosemicarbazone complexes (H_1_Ni, H_2_Ni, H_3_Ni, H_4_Ni, H_1_Cu, H_2_Cu, H_3_Cu, and H_4_Cu) were synthesized by the reaction of 4-phenyl-thiosemicarbazide (4 mmol) and 2-aminothiazole-5-carboxaldehyde (4 mmol) in ethanol/DMSO mixture. The pH of the solution was adjusted to 5–5.5 with 1 mL of acetic acid. The solution was heated with stirring under reflux for 5 h at 80 °C. The ethanol solution (50 mL) of the salicylaldehyde derivatives (4 mmol) was added to the mixture and stirred for a further 5 h at 80 °C. 5-Fluoro-3-methylsalicylaldehyde, 5-bromo-salicylaldehyde, 3-chloro-5-fluorosalicylaldehyde, and 5-methylsalicylaldehyde were used as salicylaldehyde. The ethanol solution (5 mL) of the metal salts [nickel(ii) chloride hexahydrate & copper(ii) chloride (anhydrous)] was added dropwise to the reaction mixture and stirred by refluxing for a further 4 h at 70 °C. The mixture was evaporated at room temperature. The colored product was filtered, purified, and dried.

The structures of all heterocyclic thiosemicarbazone complexes (H_1_Ni, H_2_Ni, H_3_Ni, H_4_Ni, H_1_Cu, H_2_Cu, H_3_Cu, and H_4_Cu) were determined by spectroscopic techniques.

#### 2.2.1 H_1_Ni

Yield 0.3503 g (69%), burgundy solid, mp 192 °C. IR spectrum (KBr), *ν*, cm^−1^: 3340 (OH); 829 (H_2_O); 3025 (CH)_aro._; 1615 (CH=N); 1544 (CH=N)_tyz_; 1495 (C=C); 1210, 819 (C=S); 750 (C–S–C); 1003 (N–N); 3243 (N–H); 568 (M–O); 492 (M–N). ^1^H-NMR spectrum (400 MHz, DMSO-d6), *δ*, ppm: 11.71 (1H, s, N–NH), 10.91 (^1^H, s, Ar–OH), 8.94 (1H, s, CH=N), 6.87–7.50 (5H, m, Ar–H), 2.27 (3H, s, Ar–CH_3_). Elemental analysis found%: C 44.28; H 3.75; N 13.45; S 13.06. C_19_H_19_N_5_S_2_O_2_ClNi. Calculated, %: C 43.46; H 3.26; N 13.34; S 12.21, UV-Vis (DMSO, *ε* × 10^−4^ M), *λ*_max_, nm: 383, 663.

#### 2.2.2 H_1_Cu

Yield 0.3010 g (61%), dark burgundy solid, mp 180 °C. IR spectrum (KBr), *ν*, cm^−1^: 3345 (OH); 3078 (CH)_aro._; 1616 (CH=N); 1520 (CH=N)_tyz_; 1475 (C=C); 1207, 815 (C=S); 738 (C–S–C); 1017 (N–N); 3247 (N–H); 559 (M–O); –(M–N). Elemental analysis found%: C 47.17; H 3.19; N 14.21; S 13.04. C_19_H_16_N_5_S_2_OClCu. Calculated, %: C 46.24; H 3.27; N 14.19; S 12.99, UV-Vis (DMSO, *ε* × 10^−4^ M), *λ*_max_, nm: 441, 889.

#### 2.2.3 H_2_Ni

Yield 0.4120 g (72%), burgundy solid, mp 197 °C. IR spectrum (KBr), *ν*, cm^−1^: 3337 (OH); 770 (H_2_O); 3049 (CH)_aro._; 1603 (CH=N); 1520 (CH=N)_tyz_; 1475 (C=C); 1210, 820 (C=S); 750 (C–S–C); 1014 (N–N); 3231 (N–H); 574 (M–O); 501 (M–N). ^1^H-NMR spectrum (400 MHz, DMSO-d6), *δ*, ppm: 11.46 (1H, s, N–NH), 10.45 (1H, s, Ar-OH), 8.18 (1H, s, CH=N), 6.95–7.69 (5H, m, Ar–H). Elemental analysis found%: C 38.01; H 2.64; N 12.27; S 11.17. C_18_H_16_N_5_S_2_O_2_ClBrNi. Calculated, %: C 36.65; H 2.39; N 11.87; S 10.87, UV-Vis (DMSO, *ε* × 10^−4^ M), *λ*_max_, nm: 380, 660.

#### 2.2.4 H_2_Cu

Yield 0.3629 g (65%), dark burgundy solid, mp 185 °C. IR spectrum (KBr), *ν*, cm^−1^: 3330 (OH); 3065 (CH)_aro._; 1615 (CH=N); 1506 (CH=N)_tyz_; 1471 (C=C); 1212, 817 (C=S); 747 (C–S–C); 1011 (N–N); 3228 (N–H); 596 (M–O); 479 (M–N). Elemental analysis found%: C 38.42; H 2.22; N 12.49; S 11.37. C_18_H_13_N_5_S_2_OClBrCu. Calculated, %: C 38.72; H 2.35; N 12.54; S 11.48, UV-Vis (DMSO, *ε* × 10^−4^ M), *λ*_max_, nm: 431, 887.

#### 2.2.5 H_3_Ni

Yield 0.3222 g (59%), burgundy solid, mp 201 °C. IR spectrum (KBr), *ν*, cm^−1^: 3317 (OH); 781 (H_2_O); 3080 (CH)_aro._; 1616 (CH=N); 1503 (CH=N)_tyz_; 1456 (C=C); 1208, 821 (C=S); 751 (C–S–C); 1006 (N–N); 3219 (N–H); 562 (M–O); 490 (M–N). ^1^H-NMR spectrum (400 MHz, DMSO-d6), *δ*, ppm: 11.66 (1H, s, N–NH), 10.59 (1H, s, Ar–OH), 8.22 (1H, s, CH=N), 7.02–7.68 (5H, m, Ar–H), 2.27 (3H, s, Ar–CH_3_). Elemental analysis found%: C 38.99; H 2.67; N 12.87; S 11.66. C_18_H_15_N_5_S_2_O_2_FCl_2_Ni. Calculated, %: C 38.37; H 2.33; N 12.43; S 11.38, UV-Vis (DMSO, *ε* × 10^−4^ M), *λ*_max_, nm: 379, 661.

#### 2.2.6 H_3_Cu

Yield 0.3191 g (60%), dark burgundy solid, mp 189 °C. IR spectrum (KBr), *ν*, cm^−1^: 3321 (OH); 3078 (CH)_aro._; 1612 (CH=N); 1505 (CH=N)_tyz_; 1453 (C=C); 1205, 825 (C=S); 751 (C–S–C); 1009 (N–N); 3223 (N–H); 561 (M–O); 488 (M–N). Elemental analysis found%: C 39.95; H 2.30; N 13.21; S 12.19. C_18_H_12_N_5_S_2_OFCl_2_Cu. Calculated, %: C 40.65; H 2.27; N 13.17; S 12.06, UV-Vis (DMSO, *ε* × 10^−4^ M), *λ*_max_, nm: 428, 893.

#### 2.2.7 H_4_Ni

Yield 0.3312 g (63%), burgundy solid, mp 192 °C. IR spectrum (KBr), *ν*, cm^−1^: 3331 (OH); 792 (H_2_O); 3063 (CH)_aro._; 1621 (CH=N); 1501 (CH=N)_tyz_; 1456 (C=C); 1203, 842 (C=S); 749 (C–S–C); 1005 (N–N); 3244 (N–H); 570 (M–O); 485 (M–N). ^1^H-NMR spectrum (400 MHz, DMSO-d6), *δ*, ppm: 11.25 (1H, s, N–NH), 10.61 (1H, s, Ar-OH), 8.29 (1H, s, CH=N), 7.11–7.82 (5H, m, Ar–H). Elemental analysis found%: C 43.55; H 3.71; N 13.51; S 12.49. C_19_H_18_N_5_S_2_O_2_FClNi. Calculated, %: C 42.02; H 2.97; N 12.90; S 11.81, UV-Vis (DMSO, *ε* × 10^−4^ M), *λ*_max_, nm: 377, 659.

#### 2.2.8 H_4_Cu

Yield 0.2813 g (55%), dark burgundy solid, mp 187 °C. IR spectrum (KBr), *ν*, cm^−1^: 3325 (OH); 3078 (CH)_aro._; 1616 (CH=N); 1503 (CH=N)_tyz_; 1456 (C=C); 1208, 842 (C=S); 753 (C–S–C); 1015 (N–N); 3241 (N–H); 575 (M–O); 504 (M–N). Elemental analysis found%: C 44.79; H 2.99; N 13.21; S 13.01. C_19_H_15_N_5_S_2_OFClCu. Calculated, %: C 44.62; H 2.96; N 13.69; S 12.54, UV-Vis (DMSO, *ε* × 10^−4^ M), *λ*_max_, nm: 429, 891.

### 2.3. Antimicrobial assay

Gram (+) bacteria, Gram (–) bacteria and yeast used in the antimicrobial study are as follows, respectively: (*Micrococcus luteus* ATCC9341, *Staphylococcus epidermidis* ATCC12228, *Bacillus cereus* RSKK863), (*Pseudomonas aeroginosa* ATCC27853, *Klebsiella pneumonia* ATCC27853, *Enterobacter aerogenes* ATCC51342, *Salmonella typhi* H NCTC9018394, *Shigella dysenteria* NCTC2966, *Proteus vulgaris* RSKK96026) and (*Candida albicans* Y-1200-NIH). For the antimicrobial assay, all heterocyclic thiosemicarbazone complexes (H_1_Ni, H_2_Ni, H_3_Ni, H_4_Ni, H_1_Cu, H_2_Cu, H_3_Cu, and H_4_Cu) were screened against these disease agent pathogens by the well-diffusion methods.^29–31^ In this method, it was determined that DMSO, used as solvent control, did not show antimicrobial activity against the tested organisms. As a first step, all heterocyclic thiosemicarbazone complexes were solved (3.5 μg mL^−1^) in DMSO, and all pathogenic microorganisms were incubated in Nutrient Broth agar (10^6^ CFU mL^−1^) for 24 h at 37 °C. As a second step, these cultures were then homogenized by adding them to Mueller–Hinton Agar (MHA) cooled to 45 °C, and they were poured into sterile Petri dishes and cooled. Afterward, wells of 6 mm diameter were pierced in these agars, and the heterocyclic thiosemicarbazone complexes were added. Finally, the plates were incubated in an oven (at 37 °C, 24 h), the inhibition zone of all heterocyclic compounds was measured, and then the average of the activity values performed with two repetitions was taken. As a third second, selected disease agent pathogens were compared with standard antibiotics. For this purpose, Ampicillin (AMP10), Sulphamethoxazole (SXT25), Amoxicillin (AMC30), and Kanamycin (K30) antibiotics were used for Gram (+) and Gram (–) bacteria, and Nystatin (NYS100) antibiotic was used for yeast.

## 3 Results

### 3.1. Characterization of heterocyclic compounds containing thiosemicarbazone

Some physical properties, analytical, and organic elemental analysis data of all heterocyclic thiosemicarbazone Ni^2+^ and Cu^2+^ complexes are presented in Table 1. It was defined that the elemental analyses and the chemical formulas of all heterocyclic thiosemicarbazone complexes were compatible.

**Table 1:** Some physical properties, analytical and organic elemental analysis data of heterocyclic thiosemicarbazone complexes^a^

Symbol	Chemical formula (*M*_w_, g mol^−1^)	Colour conductance (μS cm^−1^)	M. p. (°C)	Elemental analysis calculated (found)%			
			*μ*_eff_ (BM)	C	H	N	S
H_1_Ni	C_19_H_17_N_5_S_2_OCl_2_Ni	Burgundy	192	44.95	3.77	13.80	12.63
	(525.09)	35.6	D	(43.46)	(3.26)	(13.34)	(12.21)
H_1_Cu	C_19_H_16_N_5_S_2_OClCu	Dark burgundy	180	46.24	3.27	14.19	12.99
	(493.49)	15.4	1.93	(47.17)	(3.19)	(14.21)	(13.04)
H_2_Ni	C_18_H_14_N_5_S_2_OCl_2_BrNi	Burgundy	197	37.76	2.82	12.23	11.20
	(586.86)	2.5	D	(36.65)	(2.39)	(11.87)	(10.87)
H_2_Cu	C_18_H_13_N_5_S_2_OClBrCu	Dark burgundy	185	38.72	2.35	12.54	11.48
	(558.36)	15.6	1.81	(38.42)	(2.22)	(12.49)	(11.37)
H_3_Ni	C_18_H_13_N_5_S_2_OFCl_3_Ni	Burgundy	201	39.59	2.77	12.83	11.74
	(563.50)	21.8	D	(38.37)	(2.33)	(12.43)	(11.38)
H_3_Cu	C_18_H_12_N_5_S_2_OFCl_2_Cu	Dark burgundy	189	40.65	2.27	13.17	12.06
	(531.89)	17.0	2.11	(39.95)	(2.30)	(13.21)	(12.19)
H_4_Ni	C_19_H_16_N_5_S_2_OFCl_2_Ni	Burgundy	192	43.41	3.45	13.32	12.20
	(543.48)	9.0	D	(42.02)	(2.97)	(12.90)	(11.81)
H_4_Cu	C_19_H_15_N_5_S_2_OFClCu	Dark burgundy	187	44.62	2.96	13.69	12.54
	(511.48)	55.7	1.98	(44.79)	(2.99)	(13.21)	(13.01)

a D: diamagnetic.

IR spectra data of all heterocyclic thiosemicarbazone Ni^2+^ and Cu^2+^ complexes are presented in Table 2 and are shown in Fig. S1.† In the infrared spectra of all complexes, absorption bands were observed in 1003–1017 cm^−1^ and 3219–3247 cm^−1^ ranges, which were assigned to the *ν*(N–N) and *ν*(N–H) vibrations, respectively. The stretching vibrations of *ν*(CH=N) belonging to the azomethine groups obtained by the condensation reaction of aldehydes and amines, were observed in the 1603–1621 cm^−1^ and 1501–1544 cm^−1^ ranges, respectively. The absorption bands assigned to *ν*(C=C) and *ν*(CH) of the aromatic ring were observed in the 1453–1495 cm^−1^ and 3025–3080 cm^−1^ ranges, respectively. The stretching vibrations of *ν*(C–S–C) belonging to the thiazole groups were determined in the 738–753 cm^−1^ region. The stretching vibrations of *ν*(C=S) vibrations occurred in the 815–842 cm^−1^ and 1203–1212 cm^−1^ ranges, respectively. Additionally, the observation of bands in the ranges 479–504 cm^−1^ and 559–596 cm^−1^ was due to the stretching vibrations of *ν*(M–N) and *ν*(M–O). ^32^ These bands indicate the coordination of ligands to metal centers.

**Table 2:** FT-IR spectra data of heterocyclic thiosemicarbazone complexes

Symbol	*ν*(OH)	*ν*(CH)_aro._/*ν*(C=C)	*ν*(CH=N)/*ν*(CH=N)_tyz_	*ν*(C=S)	*ν*(C–S–C)	*ν*(N–N)/*ν*(N–H)	*ν*(M–O)/*ν*(M–N)
H_1_Ni	3340	3025	1615	1210	750	1003	—
		1495	1544	819		3243	492
H_1_Cu	—	3078	1616	1207	738	1017	559
		1475	1520	815		3247	490
H_2_Ni	3337	3049	1603	1210	750	1014	—
		1475	1520	820		3231	501
H_2_Cu	—	3065	1615	1212	747	1011	596
		1471	1506	817		3228	479
H_3_Ni	3317	3080	1616	1208	751	1006	—
		1456	1503	821		3219	490
H_3_Cu	—	3078	1612	1205	751	1009	561
		1453	1505	825		3223	488
H_4_Ni	3331	3063	1621	1203	749	1005	—
		1456	1501	842		3244	485
H_4_Cu	—	3078	1616	1208	753	1015	575
		1456	1503	842		3241	504

^1^H-NMR spectra data of all heterocyclic thiosemicarbazone Ni^2+^ complexes are presented in Table 3 and are shown in Fig. S2.† In the spectra of all complexes, NH protons (N–NH) appeared in the range of 1066–1171 ppm. Unsymmetric azomethine (CH=N) peaks obtained by the condensation reaction of aldehydes and amines were observed in the 8.18–8.94 ppm region. The phenolic protons (Ar–OH) and aromatic protons (Ar–H) occurred in the regions 10.45–10.91 ppm and 6.87–7.82 ppm, respectively. In addition, for H_1_Ni and H_3_Ni, the methyl protons (Ar–CH_3_) were also detected at 2.27 ppm.^33^

**Table 3:** ^1^H-NMR chemical shift (ppm) of heterocyclic thiosemicarbazone Ni(ii) complexes

Compound	N–NH	Ar–OH	CH=N	Ar–H	Ar–CH_3_
H_1_Ni	11.71	10.91	8.94	6.87–7.50	2.27
H_2_Ni	11.46	10.45	8.18	6.95–7.69	—
H_3_Ni	10.66	10.59	8.22	7.02–7.68	2.27
H_4_Ni	11.25	10.61	8.29	7.11–7.82	—

TGA analysis data of all heterocyclic thiosemicarbazone Ni^2+^ and Cu^2+^ complexes are presented in Table 4 and are shown in Fig. S3.† Thermal decomposition curves of nickel complexes show that H_1_Ni and H_2_Ni exhibited two-stage weights, while H_3_Ni and H_4_Ni exhibit three-stage weights. The thermal degradation curves of copper complexes show that H_2_Cu exhibited two-stage weights, H_1_Cu and H_3_Cu exhibited three-stage weights, and H_4_Cu exhibited four-stage weights. The residues at the end of the decomposition for all complexes were determined in the range of 2.12–6.533.41–12.57% and indicated NiO and CuO, respectively.

**Table 4:** TGA and UV-Vis data of heterocyclic thiosemicarbazone complexes

Compound	Charge transfer transition (nm)	d–d transition (nm)	Step	*T*_i_ (°C)	*T*_f_ (°C)	Residue mass at 800 °C (wt%)
H_1_Ni	383	663	1st	115.02	261.31	3.46
			2nd	320.89	408.36	
			3rd	437.02	624.15	
H_1_Cu	441	889	1st	106.89	166.67	3.41
			2nd	223.70	386.71	
			3rd	471.22	644.75	
H_2_Ni	380	660	1st	110.14	208.30	6.05
			2nd	214.18	391.47	
			3rd	488.93	670.19	
H_2_Cu	431	887	1st	121.79	387.13	5.32
			2nd	494.24	678.22	
H_3_Ni	379	661	1st	158.26	342.89	6.53
			2nd	410.75	709.38	
H_3_Cu	428	893	1st	102.86	203.77	3.84
			2nd	253.80	422.66	
			3rd	482.08	691.43	
H_4_Ni	377	659	1st	111.95	351.47	2.12
			2nd	394.44	620.19	
H_4_Cu	429	891	1st	101.57	200.36	12.17
			2nd	267.10	462.03	
			3rd	490.31	544.63	
			4th	553.66	687.51	

UV-Vis spectra data of all heterocyclic thiosemicarbazone Ni^2+^ and Cu^2+^ complexes are presented in Table 4. The electronic spectra of all nickel and copper complexes showed two main bands. π → π* transitions of the aromatic ring and n → π* transitions of the imine group were observed in the ranges of 231–252 nm and 323–327 nm, respectively. In the electronic spectra of nickel complexes, the absorption bands that appeared in the 377–383 nm range were assigned to metal–ligand charge transfer transitions. The bands observed in the 659–663 nm range were assigned to the d–d transitions, which is compatible with the Ni^2+^ square planar geometry.^34^ In the electronic spectra of copper complexes, the absorption bands observed in the 428–441 nm range were assigned to intra-ligand charge transfer transitions.^35^ The bands that appeared in the 887–893 nm range were assigned to the d–d transitions, which is compatible with the Cu(ii) tetrahedral geometry.^36^

The magnetic moments of heterocyclic thiosemicarbazone Ni^2+^ complexes were determined diamagnetic, confirming the existence of square planar geometry.^37^ The magnetic moments of heterocyclic thiosemicarbazone Cu^2+^ complexes were observed in the range of 1.81–2.11 B.M., indicating the presence of one unpaired electron in the Cu^2+^ ion.^38^ The paramagnetic behavior confirms the existence of tetrahedral geometry.^39^

The molar conductance of heterocyclic thiosemicarbazone Ni^2+^ and Cu^2+^ complexes was determined in the range 2.5–35.6 μS cm^−1^ and 15.4–55.7 μS cm^−1^, respectively, confirming the non-electrolyte nature of all complexes in DMSO solution.^40^

**Fig. 3:**
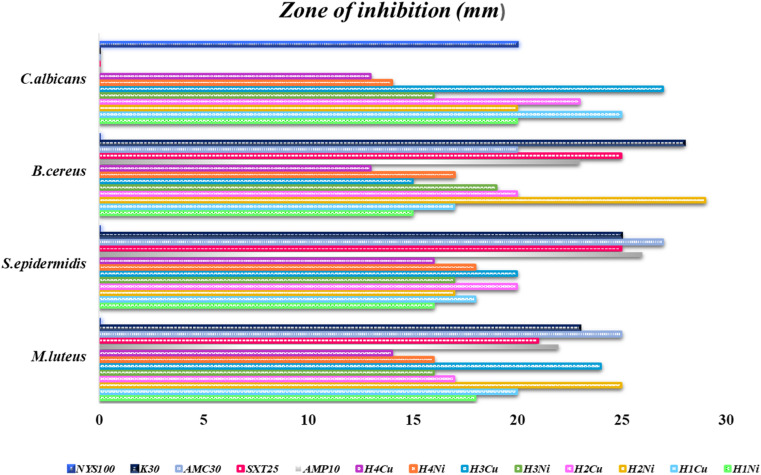
Graphical illustration of Gram (+) pathogenic bacterial species, and standard antibiotics.

**Fig. 4:**
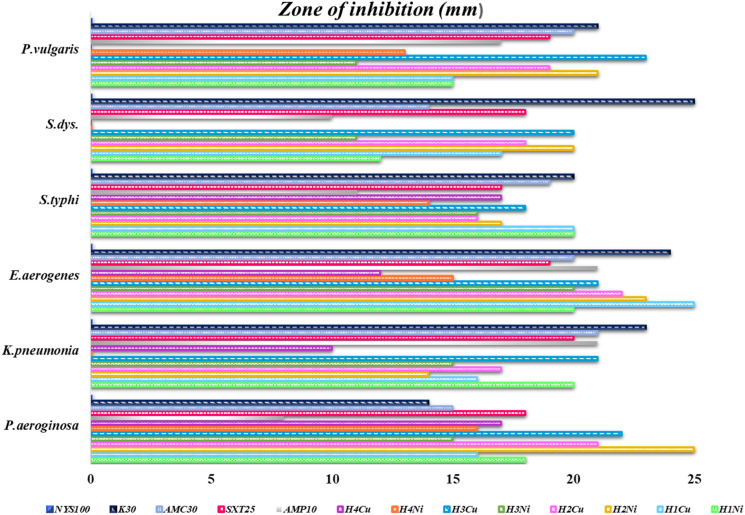
Graphical illustration of Gram (–) pathogenic bacterial species, and standard antibiotics.

**Fig. 5:**
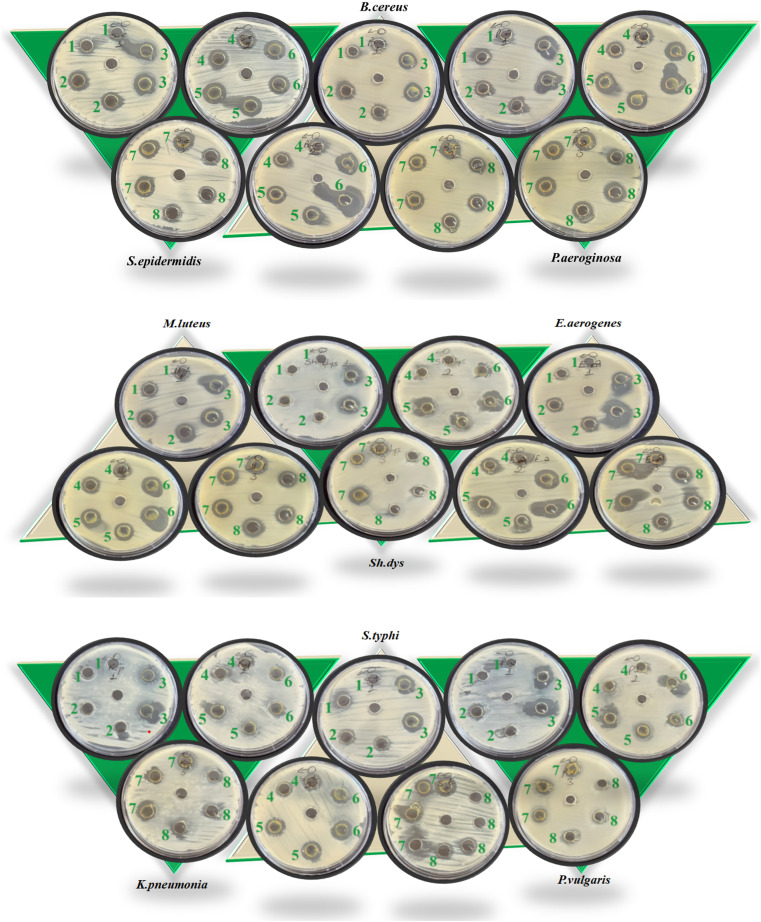
Photographs of inhibition zones (mm) of some Gram (+) and Gram (–) bacteria.

The graphical illustration and the photographs of inhibition regions of pathogenic bacterial species are presented in Fig. 3–5, respectively. The heterocyclic thiosemicarbazone Ni^2+^ and Cu^2+^ complexes' were tested *in vitro* against selected disease-causing pathogenic bacteria and yeast to determine their antibacterial and antifungal activities. The yeast and pathogens were compared to the standard antibiotics and anticandidal. The results showed that the compounds had different antimicrobial effects against Gram-positive bacteria (*M. luteus*, *S. epidermidis*, *B. cereus*), Gram-negative bacteria (*P. aeroginosa*, *K. pneumonia*, *E. aerogenes*, *S. typhi* H, *S. dysenteria*, *P. vulgaris*) and yeast (*C. albicans*). The heterocyclic thiosemicarbazone Ni^2+^and Cu^2+^ complexes generally exhibited more potent antibacterial capacity against Gram (–) than Gram (+) bacteria. H_2_Ni (29 mm) complex showed more higher inhibitory effects than all standard antibiotics against the Gram (+) bacteria *B. cereus*. This pathogen causes certain infections such as vomiting and eye.^41^ H_3_Cu and H_2_Ni complexes (24 mm and 25 mm, respectively) demonstrated higher activity than the standard antibiotics (except AMC30) against the Gram (+) bacteria *M. luteus*. This pathogen causes bloodstream infections.^42^ Among Gram (–) bacteria, all heterocyclic thiosemicarbazone complexes (16–25 mm) showed significant antibacterial activities against *P. aeroginosa* and *S. typhi* H compared to the standard antibiotics. *S. typhi* H bacteria causes typhoid.^43^ H_2_Ni (25 mm), H_2_Cu (21 mm), H_3_Cu (22 mm) complexes showed a more potent inhibitory impact than all standard antibiotics against *P. aeroginosa*. This bacteria causes urinary tract infections.^44^ H_3_Cu (23 mm) demonstrated a potent antibacterial activity than all standard antibiotics against *P. vulgaris*. This pathogen causes certain infections, such as meningitis, diarrhea, and abscesses.^45^ H_1_Cu (25 mm) showed more higher activity than all standard antibiotics against *E. aerogenes*. This pathogen causes infections in the urinary and respiratory tracts.^46^ Additionally, the antifungal effects of the heterocyclic thiosemicarbazone Ni^2+^ and Cu^2+^ complexes were compared to the standard Nystatin antibiotic against yeast *C. albicans*. This fungus causes gastrointestinal tract infections and bloodstream infections.^47^ Cu^2+^ complexes generally exhibited a more potent effect than Ni^2+^ complexes. H_3_Cu (27 mm), H_1_Cu (25 mm), and H_2_Cu (23 mm) complexes showed much stronger antifungal activity than Nystatin. H_1_Ni (20 mm) and H_2_Ni (20 mm) complexes demonstrated as much inhibitory impact as standard antibiotics.

In conclusion, the heterocyclic thiosemicarbazone Ni^2+^ and Cu^2+^ complexes were determined to have high or moderate antifungal and antibacterial activity. It was observed that the antibacterial and antifungal activities of thiosemicarbazone compounds, including electron-withdrawing groups (–Br/–Cl/–F), were more effective than the thiosemicarbazone compounds, including electron-donating group (–CH_3_). The synthesized thiosemicarbazone compounds showed very good antibacterial and antifungal properties because they contain thiazole rings containing N and S heteroatoms, nickel and copper metal ions with chelation ability, and asymmetric diimine groups that impart biological activity. This situation is consistent with similar research results.^24,48,49^ The antimicrobial activities vary depending on the presence of metal ions, the position of the substituent on the ring, and the presence of terminal groups (–CH_3_, –Ph, –F, –Cl, –Br, *etc.*). Increased and/or decreased biological activities have been reported for compounds.^27^ The antimicrobial activities increase and/or decrease depending on the presence of metal ions, the position of the substituent on the ring, and the presence of terminal groups (–CH_3_, –Ph, –F, –Cl, –Br, *etc.*).

Ni^2+^ complexes generally showed stronger antibacterial activity than Cu^2+^ complexes. Especially the H_2_Ni complex exhibited a higher inhibitory effect.

## 4 Conclusions

In this study, novel α-N-heterocyclic thiosemicarbazone compounds were synthesized using the template method and were characterized by different spectroscopic techniques. Based on the spectral studies, it was determined that Ni^2+^ complexes had a square planar geometry, while Cu^2+^ complexes had tetrahedral geometry. The biological activities of heterocyclic thiosemicarbazone compounds were evaluated against disease-causing pathogens using the well-diffusion method. All heterocyclic thiosemicarbazone complexes were observed to have high or moderate antimicrobial activity. The synthesized thiosemicarbazone compounds containing asymmetric azomethine groups exhibited different biological activities depending on the presence of metal ions and the presence of terminal groups. The heterocyclic thiosemicarbazone Ni^2+^ and Cu^2+^ complexes exhibited more potent impacts against different Gram-negative bacterial strains as potential antibacterial agents. The heterocyclic thiosemicarbazone Ni^2+^ complexes showed more potent impacts against yeast as potential antifungal agents. According to high/or moderate biological activity results, the synthesized novel α-N-heterocyclic thiosemicarbazone compounds can be recommended as potent inhibitors in diverse pharmaceutical, biological, medicinal, biomedical, *etc.* applications.

## Data availability

The data supporting this article have been included in the main article and the ESI.†

## Author contributions

D. Nartop designed the experiments and contributed to the interpretation of the results. E. Hasanoğlu Özkan carried out the experiments depend on synthesis. D. Nartop and E. Hasanoğlu Özkan performed visualization, formal analysis, writing – original draft, writing-review, and editing, H. Ogutcu carried out the antimicrobial experiments and assessments. N. Kurnaz Yetim and İ. Özdemir contributed to conceptualization and review. Each author contributed to the final manuscript and discussed the findings.

## Conflicts of interest

There are no conflicts to declare between the authors.

## Supplementary Material

RA-014-D4RA04002C-s001

## References

[cit1] Bandyopadhyay D. and Banik B. K., Microwave-induced Synthesis of Heterocycles of Medicinal Interests: In Green Synthetic Approaches for Biologically Relevant Heterocycles, Elsevier Inc., 2015, vol. 1, pp. 517–557

[cit2] Ram V. J. , Sethi A., Nath M. and Pratap R., The Chemistry of Heterocycles: Nomenclature and Chemistry of Three to Five Membered Heterocycles, Elsevier, 2019

[cit3] Taylor P. A., Robinson R. P., Fobian Y. M., Blakemore D. C., Jones L. H., Fadeyi O. (2016). Modern advances in heterocyclic chemistry in drug discovery. Org. Biomol. Chem..

[cit4] dos Santos G. C., Martins L. M., Bregadiolli B. A., Moreno V. F., da Silva-Filho L. C., da Silva B. H. S. T. (2021). Heterocyclic compounds as antiviral drugs: synthesis, structure-activity relationship and traditional applications. J. Heterocycl. Chem..

[cit5] Palaniappan S. P. (2022). Pharmacological role of heterocyclic compounds in the treatment of Alzheimer's disease: A Review. J. Phytopharm..

[cit6] Pearce S. (2017). The importance of heterocyclic compounds in anti-cancer drug design. Drug Discovery World.

[cit7] Argibay-Otero S., Carballo R., Vázquez-López E. M. (2023). Coordination chemistry of potentially S,N,Npy-Tridentate thiosemicarbazones with the {Re(CO)_3_}^+^ fragment and formation of hemiaminal derivatives. Inorg. Chem..

[cit8] Seena E. B., Sithambaresan M., Vasudevan S. (2020). *et al.*, Structural and spectral characterization of Cu(II) complexes of N(4)-substituted thiosemicarbazones derived from 2-hydroxyacetophenone: Crystal structure of a dinuclear Cu(II) complex. J. Chem. Sci..

[cit9] Matesanz A. I., Souza P. (2013). Unprecedented Pt(II) complex of an asymmetric 2,6-diacetylpyridine bis(4N-substituted thiosemicarbazone) ligand. Inorg. Chem. Commun..

[cit10] Muleta F., Eswaramoorthy T. A. R. (2019). A Review on synthesis, characterization methods and biological activities of semicarbazone, thiosemi-carbazone and their transition metal complexes. J. Nat. Sci. Res..

[cit11] Farrell N. (2002). Biomedical uses and applications of inorganic chemistry. Coord. Chem. Rev..

[cit12] West D. , Padhye S. and Sonawane P., in Structure and Bonding, Springer-Verlag, New York, 1991, vol. 76, pp. 1–49

[cit13] Basuli F., Peng S. M., Bhattacharya S. (2000). Unusual coordination mode of thiosemicarbazone ligands. a search for the origin. Inorg. Chem..

[cit14] Fernández-Fariña S., Velo-Heleno I., Martínez-Calvo M., González-Noya A. M., Pedrido R. (2022). Design, synthesis and structural characterization of a novel asymmetric hydrazone-thiosemicarbazone ligand with the aim of obtaining ınteresting metallosupramolecular architectures. ChemistrySelect.

[cit15] Kirtani D. U., Ghatpande N. S., Suryavanshi K. R., Kulkarni P. P., Kumbhar A. A. (2021). Fluorescent copper(II) complexes of asymmetric bis(thiosemicarbazone)s: Electrochemistry, cellular uptake and antiproliferative activity. ChemistrySelect.

[cit16] Özdemir N., Şahin M., Bal-Demirci T., Ülküseven B. (2011). The asymmetric ONNO complexes of dioxouranium(VI) with N1,N4-diarylidene-S-propyl-thiosemicarbazones derived from 3,5-dichlorosalicylaldehyde: Synthesis, spectroscopic and structural studies. Polyhedron.

[cit17] Varadinova T., Kovala-Demertzi D., Rupelieva M., Demertzis M., Genova P. (2001). Antiviral activity of platinum (II) and palladium (II) complexes of pyridine-2-carbaldehyde thiosemicarbazone. Acta Virol..

[cit18] Pandeya S. N., Dimmock J. R. (1993). Recent evaluations of thiosemicarbazones and semicarbazones and related compounds for antineoplastic and anticonvulsant activities. Pharmazie.

[cit19] Kasuga N. C., Sekino K., Kuomo C., Shimada N., Ishikawa M., Nomiya K. (2001). Synthesis, structural characterization and antimicrobial activities of 4- and 6-coordinate nickel (II) complexes with three thiosemicarbazones and semicarbazone ligands. J. Inorg. Biochem..

[cit20] Sharma R., Agarwal S. K., Rawat S., Nagar M. (2006). Synthesis, characterization and antibacterial activity of some transition metal cis 3,7-dimet-hyl-2,6 octadiensemicarbazone complexes. Transition Met. Chem..

[cit21] Alshater H., Al-Sulami A. I., Aly S. A., Abdalla E. M., Sakr M. A., Hassan S. S. (2023). Antitumor and antibacterial activity of Ni(II), Cu(II), Ag(I), and Hg(II) complexes with ligand derived from thiosemicarbazones: Characterization and theoretical studies. Molecules.

[cit22] Datta S., Seth D. K., Gangopadhyay S., Karmakar P., Bhattacharya S. (2012). Nickel complexes of some thiosemicarbazones: Synthesis, structure, catalytic properties and cytotoxicity studies. Inorg. Chim. Acta.

[cit23] Renade S. S., Panday V. K. (1984). Transition metals in human cancer II. Sci. Total Environ..

[cit24] Polo-Cerón D. (2019). Cu(II) and Ni(II) complexes with new tridentate NNS thiosemicarbazones: synthesis, characterisation, DNA interaction, and antibacterial activity. Bioinorg. Chem. Appl..

[cit25] Karlsson H., Fryknäs M., Strese S., Gullbo J., Westman G., Bremberg U., Sjöblom T., Pandzic T., Larsson R., Nygren P. (2017). Mechanistic characterization of a copper containing thiosemicarbazone with potent antitumor activity. Oncotarget.

[cit26] Fraústo da Silva J. J. R. and Williams R. P., The biological chemistry of the elements, Chap. 15: The Inorganic Chemistry of Life, Oxónio, Clarendon Press, 2nd edn, 1991

[cit27] Shivhare S., Gautam M. D. (2011). Electrochemical study of complexes of Cu(II) and Ni(II) with thiosemicarbazone. J. Chem. Pharm. Res..

[cit28] Bult A. , Metal Ions in Biological Systems, ed. Sigel, H., Marcel Dekker, New York, 1983, vol. 16, pp. 261–263

[cit29] Ülke E., Hasanoğlu Özkan E., Nartop D., Ogutcu H. (2022). New antimicrobial polymeric microspheres containing azomethine. J. Inorg. Organomet. Polym..

[cit30] Amutha T., Rameshbabu M., Sasi Florence S., Senthilkumar N., Vetha Potheher I., Prabha K. (2019). Studies on structural and optical properties of pure and transition metals (Ni, Fe and co-doped Ni-Fe) doped tin oxide (SnO_2_) nanoparticles for anti-microbial activity. Res. Chem. Intermed..

[cit31] Nartop D., Hasanoğlu Özkan E., Öğütçü H., Kurnaz Yetim N. (2024). Synthesis and in vitro biological assessments of novel thiazole-based thiosemicarbazone complexes. Duzce University Journal of Science and Technology.

[cit32] Alshater H., Al-Sulami A. I., Aly S. A., Abdalla E. M., Sakr M. A., Hassan S. S. (2023). Antitumor and antibacterial activity of Ni(II), Cu(II), Ag(I), and Hg(II) complexes with ligand derived from thiosemicarbazones: Characterization and theoretical studies. Molecules.

[cit33] Silverstein R. M. and Webster F. X., Spectrometric Identification of Organic Compounds, John Wiley & Sons, New York, USA, 6th edn, 1998, vol. 11, pp. 160–166

[cit34] Maurya V. K., Prasad L. B., Singh A., Shiv K., Prasad A. (2022). Synthesis, spectroscopic characterization, biological activity, and conducting properties of functionalized Ni(II) dithiocarbamate complexes with solvent extraction studies of the ligands. J. Sulfur Chem..

[cit35] Hazra M., Dolai T., Pandey A., Dey S. K., Patra A. (2014). Synthesis and characterisation of Copper(II) complexes with tridentate NNO functionalized ligand: density function theory study, DNA binding mechanism, optical properties, and biological application. Bioinorg. Chem. Appl..

[cit36] Tella A. C., Obaleye J. A. (2009). Copper(II) complexes of 4, 4- Diaminodiphenylsulphone: Synthesis, characterization and biological studies. E-J. Chem..

[cit37] Atasever Arslan B., Kaya B., Şahin O., Baday S., Saylan C. C., Ülküseven B. (2021). The iron(III) and Nickel(II) complexes with tetradentate thiosemicarbazones: Synthesis, experimental, theoretical characterization, and antiviral effect against SARS-CoV-2. J. Mol. Struct..

[cit38] Gavali L. V., Mohammed A. A., Al-Ogaili M. J. K., Gaikwad S. H., Kulkarni M., Das R., Ubale P. A. (2024). Novel terephthalaldehyde bis(thiosemicarbazone) Schiff base ligand and its transition metal complexes as antibacterial Agents: Synthesis, characterization and biological investigations. Results Chem..

[cit39] Angelusiu M. V., Barbuceanu S. F., Draghici C., Almajan G. L. (2010). New Cu(II), Co(II), Ni(II) complexes with aroyl-hydrazone based ligand: Synthesis, spectroscopic characterization and in vitro antibacterial evaluation. Eur. J. Med. Chem..

[cit40] Hasanoğlu Özkan E., Sarı N. (2020). Use of immobilized novel dendritic molecules as a marker for the detection of glucose in artificial urine. J. Mol. Struct..

[cit41] Macit A. Z., Hasanoğlu Özkan E., Ogutcu H., Nartop D. (2023). Synthesis and in vitro antimicrobial evaluation of novel potent bioactive heterocyclic compounds. Polycyclic Aromat. Compd..

[cit42] Kocoglu S., Hayvalı Z., Ogutcu H. (2021). A Polydentate ligand based on 2,2’-Dipyridylamine unit linked benzo-15-Crown-5; alkali and transition metal complexes; Photoresponsive ligand; antimicrobial evaluation against pathogenic microorganisms. Transition Met. Chem..

[cit43] Ulular M., Sarı N., Han F., Öğütcü H., Hasanoğlu Özkan E. (2024). Synthesis, antimicrobial, and DNA-binding evaluation of novel schiff bases containing tetrazole moiety and their Ni(II) and Pt(II) Complexes. Pharm. Chem. J..

[cit44] Nartop D., Tokmak E., Hasanoğlu Özkan E., Kızıl H. E., Öğütcü H., Ağar G., Allı S. (2020). Synthesis of novel polymers containing Schiff base as potential antimutagenic and antimicrobial agents. J. Med. Chem. Sci..

[cit45] Drzewiecka D. (2016). Significance and roles of *Proteus* spp. bacteria in natural environments. Microb. Ecol..

[cit46] Cabral J. P. S. (2010). Water microbiology. Bacterial pathogens and water. Int. J. Environ. Res. Public Health.

[cit47] Carolus H., Dyck K. V., Dijck P. V. (2019). *Candida albicans* and *Staphylococcus* species: A threatening twosome. Front. Microbiol..

[cit48] Kumar V. A., Sarala Y., Siddikha A., Vanitha S., Babu S., Reddy A. V. (2018). Synthesis, characterization antimicrobial and antioxidant activities of 2,4-dihydroxybenzaldehyde-4-phenyl-3-thiosemicarbazone (DHBPTSC) and its Pd(II), Ni(II)dppm mixed ligand and Cu(II) complex having heterocyclic bases. J. Appl. Pharm. Sci..

[cit49] Jain P., Sharma S., Kumar N., Misra N. (2020). Ni(II) and Cu(II) complexes of bidentate thiosemicarbazone ligand: Synthesis, structural, theoretical, biological studies and molecular modeling. Appl. Organomet. Chem..

