# Supplementary material for: Novel α-N-heterocyclic thiosemicarbazone complexes: synthesis, characterization, and antimicrobial of properties investigation
Source: RSC Adv. 2024 Sep 16;14(40):29308–18. doi: 10.1039/d4ra04002c (PMC11403394; doi:10.1039/d4ra04002c)
Supplement: RA-014-D4RA04002C-s001 [file RA-014-D4RA04002C-s001.pdf]

Supplementary Information (SI) for RSC Advances.
This journal is © The Royal Society of Chemistry 2024

Supporting Information

Novel α-N-heterocyclic thiosemicarbazone complexes: Synthesis, characterization, and antimicrobial of properties investigation

Dilek Nartop[a], Elvan Hasanoğlu Özkan[b,*], Hatice Öğütcü[c], Nurdan Kurnaz Yetim[d], İnci Özdemir[e]

[a]Department of Chemistry, Faculty of Arts and Sciences, Düzce University, Düzce, Türkiye ; e-mail : dileknartop@duzce.edu.tr

[b]Department of Chemistry, Faculty of Sciences, Gazi University, Ankara, Türkiye; e-mail : ehasanoglu@gazi.edu.tr

[c]Department of Field Crops, Faculty of Agriculture, Kırşehir Ahi Evran University, Kırşehir, Türkiye; e-mail : hogutcu@ahievran.edu.tr

[d]Department of Chemistry, Faculty of Arts and Sciences, Kırklareli University, Kırklareli, Türkiye; e-mail : nurdankurnazyetim@klu.edu.tr

[e]Kocaeli University, Izmit Vocational School, Property Protection and Security Department, 41285, Kocaeli, Türkiye; e-mail : inci.ozdemir@kocaeli.edu.tr

List of contents

IR spectra of the heterocyclic thiosemicarbazone complexes (**H_1Ni**, **H_2Ni**, **H_3Ni**, **H_4Ni**, **H_1Cu**, **H_2Cu**, **H_3Cu**, **H_4Cu**)……………………………………………………………………..Figure S1

^{1}H-NMR spectra of the heterocyclic thiosemicarbazone complexes (**H_1Ni**, **H_2Ni**, **H_3Ni**, **H_4Ni**, **H_1Cu**, **H_2Cu**, **H_3Cu**, **H_4Cu)**…………………..Figure S2

Figure S3. TGA spectra of the heterocyclic thiosemicarbazone complexes (**H_1Ni**, **H_2Ni**, **H_3Ni**, **H_4Ni**, **H_1Cu**, **H_2Cu**, **H_3Cu**, **H_4Cu**)………………………………………………………… Figure S3

Supplementary Material

Herein, IR, ^{1}H-NMR, and TGA spectra of the heterocyclic thiosemicarbazone complexes(**H_1Ni**, **H_2Ni**, **H_3Ni**, **H_4Ni**, **H_1Cu**, **H_2Cu**, **H_3Cu**, **H_4Cu**)

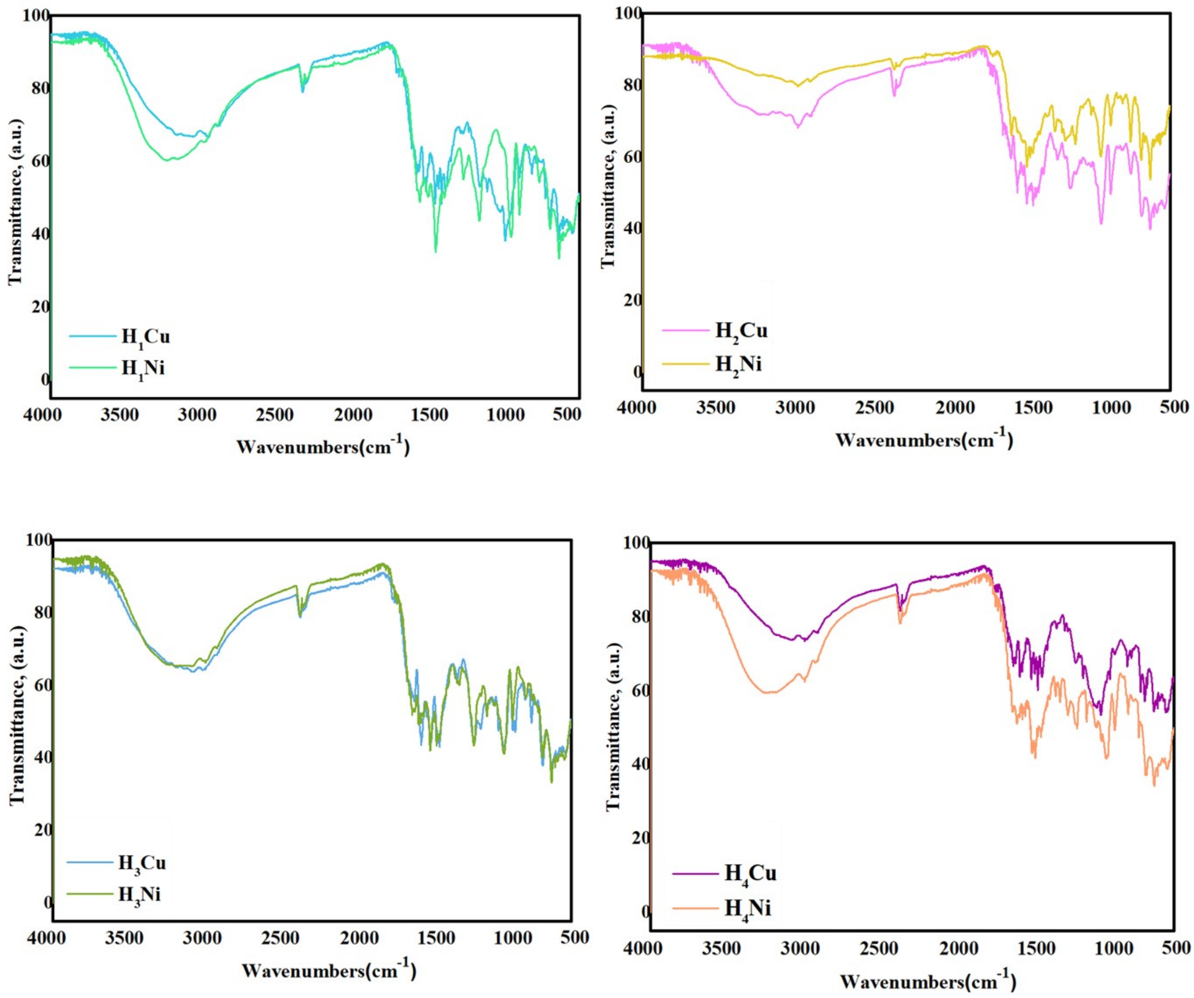

Fig. S1. IR spectra of the heterocyclic thiosemicarbazone complexes (**H_1Ni**, **H_2Ni**, **H_3Ni**, **H_4Ni**, **H_1Cu**, **H_2Cu**, **H_3Cu**, **H_4Cu**)

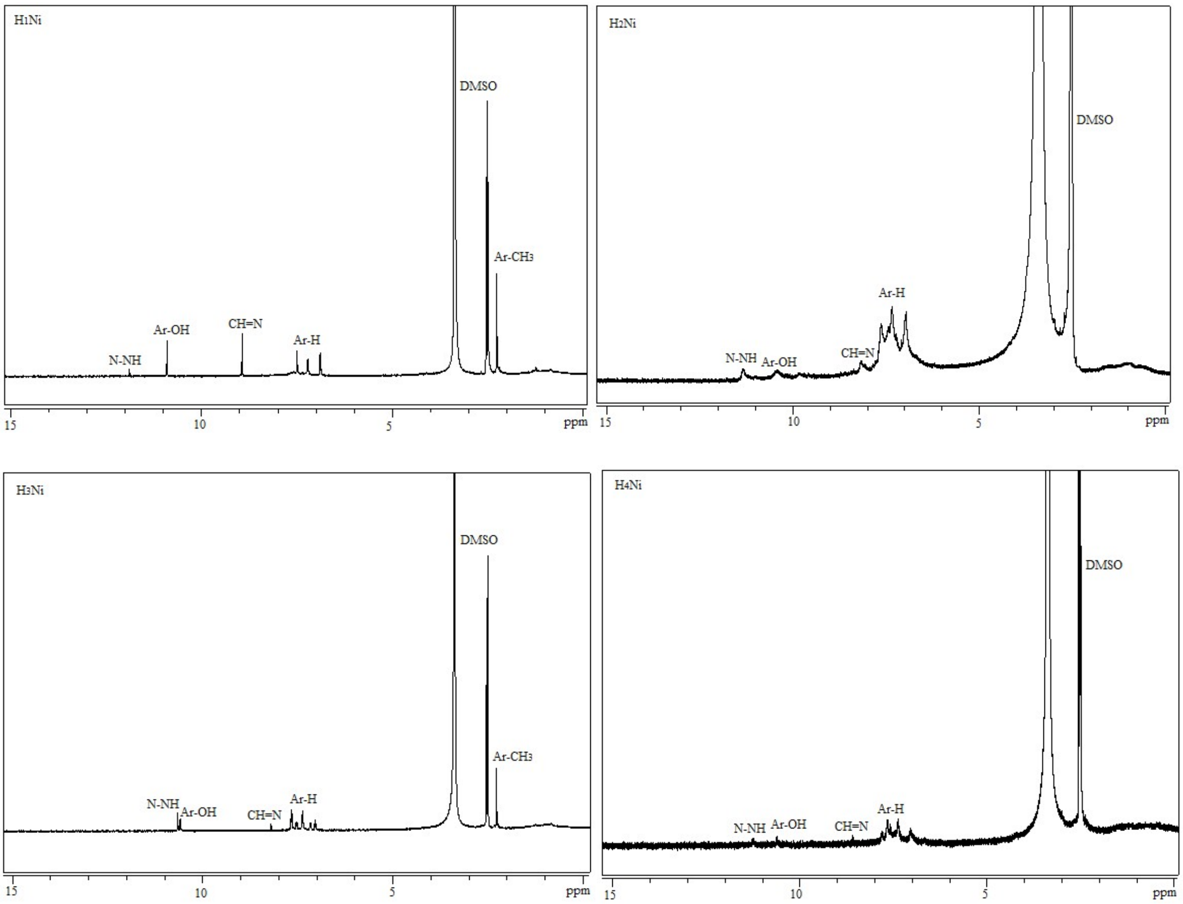

Fig. S2. ^{1}H-NMR spectra of the heterocyclic thiosemicarbazone complexes (**H_1Ni**, **H_2Ni**, **H_3Ni**, **H_4Ni**)

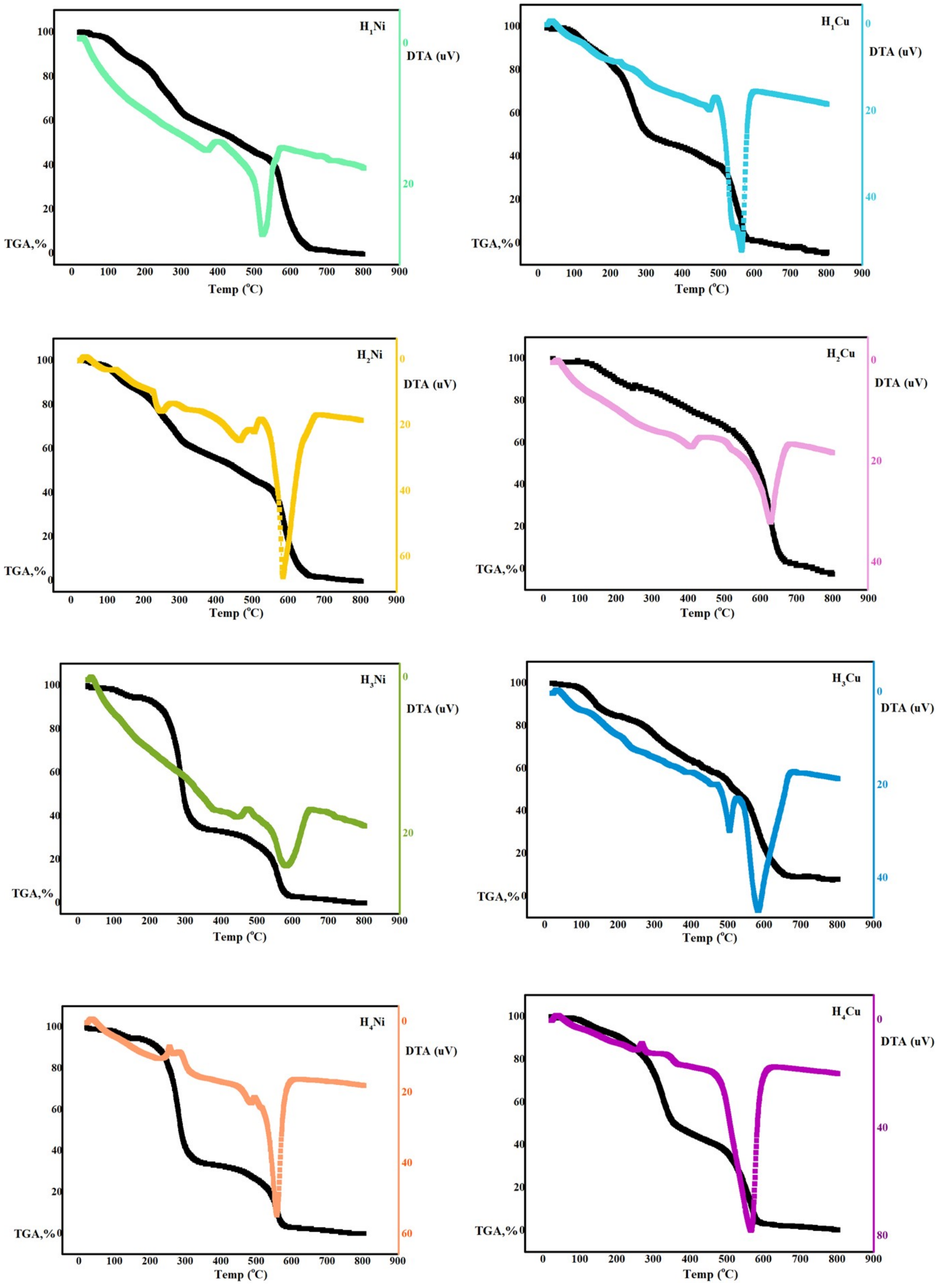

Fig. S3. TGA spectra of the heterocyclic thiosemicarbazone complexes (**H_1Ni**, **H_2Ni**, **H_3Ni**, **H_4Ni**, **H_1Cu**, **H_2Cu**, **H_3Cu**, **H_4Cu**)
